# Attention Effects on Neural Population Representations for Shape and Location Are Stronger in the Ventral than Dorsal Stream

**DOI:** 10.1523/ENEURO.0371-17.2018

**Published:** 2018-06-05

**Authors:** Anne B. Sereno, Sidney R. Lehky

**Affiliations:** 1 Department of Psychological Sciences, Purdue University, West Lafayette, IN 47907; 2 Weldon School of Biomedical Engineering, Purdue University, West Lafayette, IN 47907; 3 Computational Neurobiology Laboratory, The Salk Institute, La Jolla, CA 92037

**Keywords:** macaque monkey, object attention, population coding, spatial attention, vision

## Abstract

We examined how attention causes neural population representations of shape and location to change in ventral stream (AIT) and dorsal stream (LIP). Monkeys performed two identical delayed-match-to-sample (DMTS) tasks, attending either to shape or location. In AIT, shapes were more discriminable when directing attention to shape rather than location, measured by an increase in mean distance between population response vectors. In LIP, attending to location rather than shape did not increase the discriminability of different stimulus locations. Even when factoring out the change in mean vector response distance, multidimensional scaling (MDS) still showed a significant task difference in AIT, but not LIP, indicating that beyond increasing discriminability, attention also causes a nonlinear warping of representation space in AIT. Despite single-cell attentional modulations in both areas, our data show that attentional modulations of population representations are weaker in LIP, likely due to a need to maintain veridical representations for visuomotor control.

## Significance Statement

Disruption of attention is a fundamental characteristic of many human disorders. Although much work has focused on how attention to shape and space modulates responses at a single neuron level, that has provided little insight into how these modulations translate into effects at the population level. Similar to prior findings, we show attentional modulations at the single-cell level in both ventral and dorsal cortical areas. However, we also show that attention alters population representations of both shape and space more strongly in the ventral stream (AIT) than in the dorsal stream (LIP). This study is a first demonstration of how attention modulates the organization of high-dimensional neurophysiological representations, findings that help link cellular attentional modulations more tightly with behavior.

## Introduction

A disruption of attention is considered a key characteristic in a range of human neurologic, psychiatric, and developmental diseases such as Parkinson’s disease, schizophrenia, and autism. Attention has a variety of effects on neural activity. First, there are competitive interactions that occur when directing attention to one of multiple stimuli presented simultaneously within a single receptive field (Moran and Desimone, 1985; Motter, 1993; Luck et al., 1997; Reynolds et al., 1999; Reddy et al., 2009; Lee and Maunsell, 2010; Zhang et al., 2011). Second, attention modulates the gain of neural responses to a single stimulus within a receptive field (Colby et al., 1996; McAdams and Maunsell, 1999, 2000; Treue and Martínez-Trujillo, 1999; Reynolds et al., 2000; Martínez-Trujillo and Treue, 2002; Williford and Maunsell, 2006). Third, attention changes the statistics of stimulus responses. These include a decrease in the Fano factor (Mitchell et al., 2007; Cohen and Maunsell, 2009), a decrease in noise correlation between neurons (Cohen and Maunsell, 2009, 2011; Mitchell et al., 2009), and an increase in response correlations (e.g., γ-synchronization; Fries et al., 2001, 2008; Bichot et al., 2005; Lakatos et al., 2008; Wróbel, 2014).

In all three cases, attention increases the signal-to-noise ratio, or in other words, increases stimulus salience. Competitive effects reduce perturbations or “noise” caused by irrelevant stimuli. Increases in gain or synchrony increase the numerator of the signal-to-noise ratio, while noise attenuation decreases the denominator. The idea that attention increases stimulus salience dominates current thinking.

However, there is also a fourth class of attentional effects, one that does not involve modulating the signal-to-noise ratio, which has received less consideration. That is a shift in tuning curves caused by attention. Attention can shift receptive fields (spatial tuning curves) toward the attended location (Connor et al., 1996, 1997; Tolias et al., 2001; Womelsdorf et al., 2006, 2008; Anton-Erxleben et al., 2009; Kay et al., 2015). Attention can also cause shifts in feature tuning curves toward the attended feature characteristic (David et al., 2008; Ipata et al., 2009, 2012; Ibos and Freedman, 2014, 2016). These features include color, motion direction, shape, and orientation. Similarly, attention can also cause a shift in tuning for object categories (Çukur et al., 2013). Tuning curve shifts caused by attention change the neural representation of a stimulus, rather than increase the salience for a particular representation.

This study focuses on how attention changes stimulus representations rather than how attention increases stimulus salience. We look at these attentional effects at the population level, examining how attention to stimulus shape or attention to stimulus location across populations of cells affects the organization of multidimensional representation spaces defined by this population activity. In particular we examine (1) how attention to shape or location alters the representational discriminability or distinctiveness of different shapes or different locations (e.g., does attention magnify the distances between shapes in the neural representation space) and (2) whether attention to shape or location warps the organization of the representation space in a way that goes beyond simple magnification. Furthermore, we make a comparison of these attentional effects in a dorsal stream structure (LIP) and a ventral stream structure (AIT) under identical conditions. Such findings would provide the first insight into how single-cell attentional modulations combine to alter the population representation of shape and space and provide a first comparison across the two cortical streams of attentional changes in the representation of shape and location at the level of neural populations.

Each stimulus is represented by a point in the neural representation space. The distance between different stimuli within the representation space is an indication of how similar or how distinct the stimuli are. The goal of this study is to examine how attention modifies the relative positions of stimuli in neural representation spaces. Our analysis of the data centers on multidimensional scaling (MDS), which can be considered a form of intrinsic population decoding (Lehky et al., 2013). MDS has previously been used to visualize shape representations in neural representation space for both the dorsal (Murata et al., 2000; Romero et al., 2013) and ventral visual streams (Rolls and Tovee, 1995; Op de Beeck et al., 2001; Vogels et al., 2001; Eifuku et al., 2004; Kiani et al., 2007). In our own work we have used MDS to construct neural representation spaces for stimulus shape (Lehky and Sereno, 2007), stimulus retinal position (Sereno and Lehky, 2011a), eye position (gaze angle; Sereno et al., 2014), as well as modeling of stimulus retinal position (Lehky and Sereno, 2011) and eye position (Lehky et al., 2016). Here, we extend this work to examine how attention modulates representation spaces for stimulus shape and retinotopic location. This work also extends a previous analysis of single-cell effects of attention (Sereno and Amador, 2006).

To do this, we recorded while the monkey performed two attention tasks. In one, attention was directed to stimulus shape and in the other to stimulus location. Stimuli were physically identical in both cases. Passive fixation conditions provided baseline responses to the same stimuli for comparison with the attentional tasks.

## Materials and Methods

### Animals, animal care, and surgical procedures

Two male macaque monkeys were trained to perform the behavioral tasks described below. A scleral search eye coil, implanted before training, monitored eye position. We recorded from both LIP and AIT of each monkey. The chambers for LIP were implanted first and centered 10–12 mm lateral and 3–5 mm posterior, and the chambers for AIT were implanted after recording from LIP and were centered 18 mm anterior and 18–21 mm lateral. Details of the surgical procedures have been described earlier (Sereno and Amador, 2006; Lehky and Sereno, 2007). All experimental protocols were approved by the Baylor College of Medicine, Rutgers University, and University of Texas Animal Welfare Committees and complied with the National Institutes of Health Guidelines.

### Stimuli

**Figure 1.**
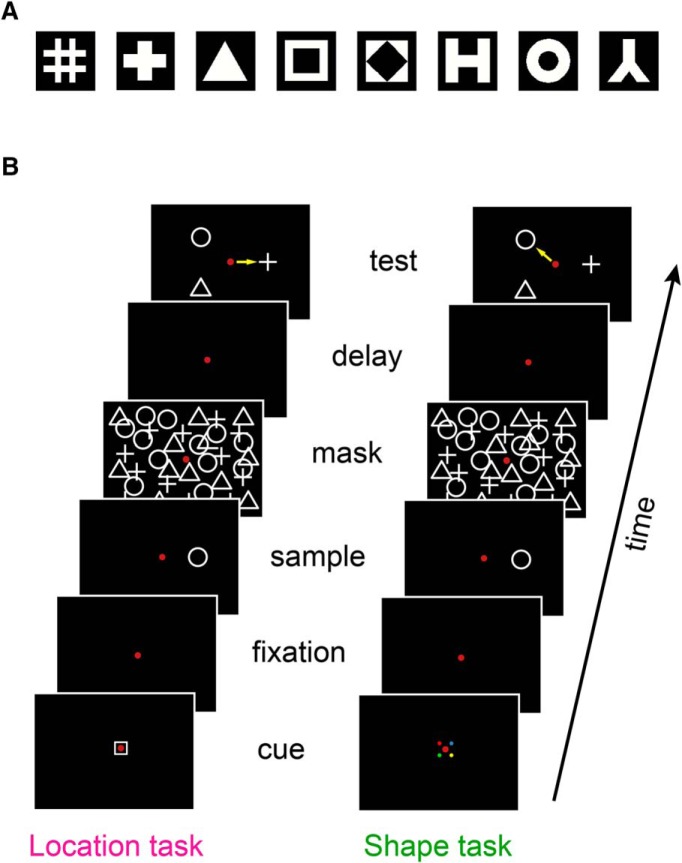
Stimuli and task. ***A***, Eight stimulus shapes used in the task. ***B***, Task sequence. A cue at start of trial indicated to the monkey which of two attention tasks to perform, either a shape attention task or a location attention task. Following the cue, a sample stimulus was shown, which consisted of a single shape presented at a particular location. Following a delay period, the test stimulus was shown, which consisted of three shapes presented at three locations. For the shape attention task, the monkey made a saccade to the test shape matching the sample shape, ignoring location. For the location attention task, the monkey made a saccade to the location matching the location of the sample stimulus, ignoring shape.

Stimuli for all tasks were chosen from among eight possible shapes (Fig. 1*A*; Sereno and Maunsell, 1998). Each shape was a simple 2D geometric form, black-and-white in color and presented on the screen against a black background. All shapes had an equal number of white pixels and fit within a square region of the same size.

### Behavioral tasks

We recorded neural responses while the monkeys performed four different behavioral tasks. These included two delayed-match-to-sample (DMTS) attention tasks, namely a shape attention DMTS task and a location attention DMTS task. A comparison of neural responses under these two tasks at a population level is the focus of this report. The other two tasks were a passive shape task (all eight shapes presented at a single location) and a passive location task (a single shape presented at eight locations), both used to characterize general response properties of each neuron without active attentional modulations.

#### Attention tasks

The two DMTS attention tasks have been described in detail in Sereno and Amador (2006) and are summarized in Figure 1*B*.

At the start of each trial the monkey was centrally fixated. A small cue surrounding the fixation point then informed the monkey which attention task to perform. Following the cue a sample stimulus was presented. The sample stimulus consisted of a single shape (selected from eight possible shapes) presented at one of eight possible eccentric locations arranged in a circle around the fixation spot. For each cell the stimulus eccentricity was always constant. For different cells across the recorded population eccentricity varied (AIT mean eccentricity 4.0°, range 10.0–2.0°; LIP mean eccentricity 11.2°, range 17.7–7.4°). Following a delay period (variable from 600 to 2700 ms), an array of test stimuli was presented. The test stimuli consisted of three shapes (again selected from the eight possible shapes) presented at three locations around the fixation spot, and at the same eccentricity as the sample.

For the shape attention task, the monkey made a saccade to the test shape matching the sample shape, ignoring location. For the location attention task, the monkey made a saccade to the location matching the location of the sample stimulus, ignoring shape. We were interested in examining attentional effects during the sample presentation period (shifted by visual latency).

The stimuli were identical for the two attention tasks. The cue at the start of the trial indicated to the monkey whether to direct attention to stimulus shape or stimulus location to successfully perform the task for a juice reward. Stimulus size ranged from 0.65° to 2.00°, increasing with eccentricity to maintain discriminability.

Catch trials typically comprised 20% of trials. Catch trials for the shape attention task did not include the sample shape in the test array, and those for the location attention task did not include the sample location. During catch trials, the monkey was rewarded for maintaining fixation on the central spot until it disappeared after an additional 1.3–1.9 s.

#### Passive shape task

Sereno and Amador (2006) have previously described this task. For this task, the stimulus shape was placed at the receptive field location producing the most effective response during the attention tasks. In each trial, the stimulus shape was randomly selected from among the same set of eight shapes used in the attention tasks. The selected shape was then presented four times in rapid succession (250–300 ms each time) before extinguishing the central fixation spot. The monkeys were rewarded for maintaining fixation on the central spot until it disappeared. Thus, over the course of multiple trials, the response of the neuron to all eight shapes was determined at the most effective location.

#### Passive location task

Sereno and Amador (2006) have also previously described this task. Each trial began with the presentation of a fixation point at the center of the visual display. Then the most effective stimulus shape for the neuron (out of the eight possibilities) was presented at one of eight peripheral locations arranged in a ring around the fixation spot, at the same eccentricities used in the attention tasks. The animal was required to make a saccade to the stimulus to obtain a juice reward. We were interested in the period after the stimulus appeared on the screen and before the monkey made a saccade toward it, when the stimulus was at one of eight possible retinal positions. Thus, over the course of multiple trials the response of the neuron at eight locations was determined for the most effective shape.

### Recording procedures

Stimuli were displayed on a 20-inch, 75-Hz CRT monitor with a resolution of 1152 × 864 pixels, placed 65 cm in front of the animal. The monitor subtended a visual angle of 27^°^ height and 36^°^ width. Beyond the monitor was a featureless 45 × 60-cm black screen (40° × 54° visual angle), which supported an electromagnetically shielded window in its center through which the animal viewed the monitor. The monkeys viewed the stimuli binocularly. Experiments were conducted in a darkened room. Action potentials were recorded extracellularly while animals performed the behavioral tasks, using either transdural tungsten (1–2 MΩ) or Pt/Ir single microelectrodes (1–2 MΩ, MicroProbes for Life Science). Electrode signals were amplified, filtered, and transformed into pulses by a window discriminator. Spike times were recorded with a resolution of 1 ms.

Histologic reconstruction in one monkey showed that the units recorded in posterior parietal cortex lay within area LIP in the lateral bank of the intraparietal sulcus. We did not distinguish between LIPd and LIPv, although cells were generally closer to the fundus than the lip of the sulcus. Units recorded in AIT were in the lower bank of the superior temporal sulcus (STS) and convexity of the middle temporal gyrus. A few perirhinal cells were included at our most anterior recording positions. Further details are given in Sereno and Maunsell (1998); Lehky and Sereno (2007)

Monkeys performed the two attention tasks while we slowly advanced the recording electrode with a hydraulic micromanipulator in search of neurons. We recorded from any neuron that was well isolated and appeared stable. After isolating a neuron, we presented a stimulus at various locations to determine the receptive field location that produced the most effective response. Typically, we tested eight locations equally spaced in a circle around the fixation spot. Then we selected three of those locations for use in the attention tasks. The three possible stimulus locations were selected so that at least one fell within the receptive field at the location that produced the most effective response at a given eccentricity. The three chosen locations were typically equally spaced at 120° intervals. We then presented all eight possible shape stimuli at the preferred location and selected three of them for the attention tasks. For most units, we chose the two shapes that produced the strongest response and the one that produced the weakest response. For most units, we then collected data with the two attention task trials intermixed. Shape and location attention task trials were intermixed either by presenting them in nine-trial blocks or randomly interleaved on a trial-by-trial basis. However, for a few units, data were collected sequentially for the two attention tasks. As long as the isolation could be maintained, we recorded with a goal of obtaining data from 216 correct matching trials [12 trials for each of nine sample conditions (three shapes by three locations) for each of two matching tasks], requiring ∼25–30 min to collect. For each cell included in data analysis we recorded a minimum of five repetitions (median 12) of each combination of sample location, sample shape, and task.

Following completion of the two attention tasks we also recorded responses during the passive shape and passive location tasks, provided the cell remained well isolated. In the passive shape task, there was a median of four repeat trials for each of the eight shapes, with four repeats of the same shape within each trial. Trial response was the average of the four repeats. For the passive location task, there was a median of 12 repeat trials of each of the eight locations.

### Data analysis

We examined attentional effects during the sample period of the DMTS tasks. As DMTS is a working memory task, our data analyses thus focused on the stimulus encoding phase of working memory, and not the retention and retrieval phases of the task. As the sample stimulus appeared by itself on an otherwise black screen (aside from the fixation spot), it was a singleton stimulus.

Data analysis focused on using MDS to compare population responses during different tasks. However, using MDS mathematically requires that every neuron in the recorded population receives an identical stimulus set, and that was not the case for the two attention tasks. Although for the two tasks both the stimulus shapes and stimulus locations were identical for each particular cell, stimulus shapes and locations varied from cell to cell. During the attention tasks, each neuron was tested with a random subset of three stimulus shapes from the eight shapes used in the passive shape task due to time constraints. Similarly, during the attention tasks each neuron was tested at a different set of only three locations roughly 120° apart along in circle centered on the fixation point, with those locations adjusted to reflect the position of that neuron’s receptive field. Completing both attention tasks with all combinations of shapes in eight locations would have required stable recording for over three hours for each cell.

The three locations in the attentional tasks approximately corresponded to three of the eight locations in the passive tasks, although they were not exactly the same. We used interpolation to estimate responses at exactly matching locations, as described below. The response interpolation was done independently for each neuron.

We calculated the interpolation as follows. In the passive task, there were eight stimulus locations at constant eccentricity arranged along a circle around fixation. In the active task, there were three stimulus locations arranged along a circle at the same eccentricity. The polar angle positions of the three active locations did not exactly match any of the polar angle positions of the eight passive locations, although they matched in eccentricity. A 1D spline interpolation was calculated along the circle of constant eccentricity using responses from the eight passive-task points to estimate passive responses at polar angles corresponding to the three points in the active task. For AIT the mean shift in polar angle during interpolation was 4.9°. In other words, on average, we estimated the neural response of a cell at a location shifted 4.9° in polar angle and 0° in eccentricity from the location of a data point. For LIP the median shift in polar angle during interpolation was 5.2°.

#### Attentional gain factors

We wanted to compare responses under the attentional tasks and the passive tasks using MDS. We therefore had to estimate attentional responses for the eight shapes and eight locations used in the passive tasks, based on the three shapes and three locations actually used in the attentional tasks, all collected from the same cell. To do that, for each cell we calculated an attentional gain factor based on responses to the three stimuli (shapes or locations) that were used in both the attentional and passive tasks. Then that gain factor was used to estimate attentional responses to the remaining stimuli that were used in the passive task but not used in the attentional task. For each cell, we determined four gain factors: (1) shape attentional gain factor when decoding population data for stimulus shape; (2) location attentional gain factor when decoding population data for stimulus shape; (3) shape attentional gain factor when decoding population data for stimulus location; and (4) location attentional gain factor when decoding population data for stimulus location.

The shape attention task required endogenous attention directed to stimulus shape, while the location attention task required endogenous attention directed to stimulus location. In contrast, the passive shape and passive location tasks were passive in the sense that they did not require deployment of endogenous object or location selective attention. Thus, taking the ratio of responses to the same stimuli in the same neuron during an attention task and a passive task allows one to define an attentional gain factor for the attention task. As all stimuli had fixed contrast at maximum value (contrast = 1.0), we treated the modulation of neural activities by attention as response gain (McAdams and Maunsell, 1999; Treue and Martínez-Trujillo, 1999) rather than contrast gain (Reynolds et al., 2000; Martínez-Trujillo and Treue, 2002).

After determining the attentional gain factors for a neuron based on the three overlapping stimuli between the attention and passive tasks, all eight stimuli (shape or location) in the passive task were then multiplied by that gain factor to estimate their responses during the attention task.

For the calculation of the attentional gain factors, a linear regression was calculated between responses to the three stimuli in the attention task and the three corresponding stimuli (out of eight stimuli) in the passive task. The regression was subject to the constraint that it passed through the origin. The slope of this regression line defined the attentional gain factor. The assumption underlying this procedure was that the effect of attention was to linearly scale the passive responses of neurons by some multiplicative factor. This assumption is supported by extensive experimental evidence for both spatial and feature attention (McAdams and Maunsell, 1999; Maunsell and Treue, 2006; Maunsell, 2015).

##### Neural representation of shape space

We derived two attentional gain factors for a given cell relative to the passive shape task. One was based on responses during the shape attention task relative to the passive shape task and the other based on responses during the location attention task relative to the passive shape task. Multiplying the passive shape data by these two attentional gain factors gave two estimates of responses to the eight shapes, one when directing attention to stimulus shape and the other when directing attention to stimulus location. Following this procedure for every cell in the recorded population produced two MDS population estimates of the neural shape representation space under different attentional conditions (shape or location).

##### Neural representation of location space

We also derived two attentional gain factors for that cell relative to the passive location task. One was based on responses during the shape attention task relative to the passive location task and the other based on responses during the location attention task relative to the passive location task. Multiplying the passive location data by these two attentional gain factors gave two estimates of responses to the eight locations, one when directing attention to stimulus shape and the other when directing attention to stimulus location. Following this procedure for every cell in the recorded population produced two MDS population estimates of the neural location representation space under the two attentional conditions.

##### Statistical power analysis

We performed a power analysis on the difference between mean vector response distances under the shape attention task and the location attention task. The power analysis was done using the MATLAB (MathWorks Inc.) sampsizepwr command in the Statistics and Machine Learning Toolbox with testtype set to z-test. Sample size was 28, which is the number of paired comparisons *n*(*n*-1)/2 for *n* = 8 shapes or locations. The null hypothesis was that there was zero mean response difference between the two attentional conditions with a standard deviation as given by our data. For the alternative hypothesis we set the mean response distance to a value that produced a statistical power of 0.8 at *p* = 0.05.

On decoding neural population data for stimulus shape, in AIT an attentional effect of 11.2% produced a power of 0.8, while in LIP an attentional effect of 6.1% produced a power of 0.8. When decoding the data for stimulus location, an attentional effect of 5.2% in AIT and 3.6% in LIP produced a power of 0.8.

Published data shows attentional effects are strong in extrastriate areas, and it is expected in mid to late stages to have attentional modulations of 20–50% (McAdams and Maunsell, 1999; Martínez-Trujillo and Treue, 2002; Williford and Maunsell, 2006). Thus, this analysis indicates that our sample size was sufficient to reveal significant statistical effects in the areas from which we were recording.

#### Multidimensional scaling (MDS)

Using MDS, it was possible to decode the data to establish a shape representation space using responses from eight shapes or decode the data to establish a location representation space using responses from eight locations. These provided estimates of neural shape representation space and neural location representation space, respectively.

The shape representation space (using MDS to decode for shape) was determined when directing attention either to stimulus shape or stimulus location. The location representation space (using MDS to decode for location) was also determined when directing attention to stimulus shape or stimulus location. Therefore, four MDS analyses under attentional conditions were performed.

Stimulus eccentricities had been adjusted for each recorded neuron to reflect the position of that neuron’s receptive field. To make these data mathematically suitable for MDS analyses when decoding for stimulus location, we only included those cells whose stimuli fell in a narrow band of eccentricities (AIT: 2–6°; LIP: 7–12°). Then, for purposes of MDS, we mathematically treated the stimulus eccentricities for all cells as if they were identically located at the average eccentricity of the population. We have previously used this averaging method for MDS analysis in (Lehky and Sereno, 2007; Sereno and Lehky, 2011a; Sereno et al., 2014).

At this point, the MDS procedure was the same as what we have done in previous studies for both stimulus shape (Lehky and Sereno, 2007) and stimulus location (Lehky and Sereno, 2011; Sereno and Lehky, 2011a; Sereno et al., 2014; Lehky et al., 2016). The first step was to define a population response vector for each stimulus. The population response vector was an ordered list of the firing rates of all neurons in the recorded population to the same stimulus. The set of neural responses forming a single response vector pooled data from multiple recording sessions.

There was one population response vector for each stimulus. When decoding for shape we had eight population response vectors, one for each of the eight shapes in our stimulus set whose responses were measured at the most effective location. When decoding for location we also had eight population response vectors, one for each of the eight locations in our stimulus set whose responses were measured using the most effective shape.

##### Response distances

To test how attention alters the response discriminability or distinctiveness of stimuli, we calculated the mean distance between population response vectors to all eight shapes or all eight locations. Each element in a response vector represented the mean response of a single neuron to the stimulus. A larger mean response distance between stimuli in the representation space indicated that the stimuli were more discriminable or distinctive. A larger mean would thus be an indication that attention caused a general expansion or magnification of the representation space.

To calculate mean response distance, we first measured the distances between all the response vectors. With our *n* = 8 stimuli (shape or location) this produced an 8 × 8 distance matrix containing *n*(*n*-1)/2 = 28 unique distances. We used a correlation distance metric, defined as *d = 1-r* where *r* is the correlation coefficient between the elements of two response vectors. Thus, response vectors to different stimuli (e.g., different shapes or different locations) that had less correlated responses across the population would have larger response distances within the representation space and be more discriminable or distinctive. Mean response distance was then determined by averaging all response distances between different stimuli. Note we measured the correlation between response vectors for the same neural population under two behavioral conditions, not correlations between individual neurons within that population.

##### MDS plots

To test whether attention could alter the organization of the neural representational space (i.e., relative positions of individual stimuli), we compared results from MDS analyses under different attentional conditions. A question here was whether attention could cause a warping of the representation space, apart from any magnification effects on the space. The distance matrix, described above in *Response distances*, also serves as the input to the MDS algorithm proper. To do MDS we used the cmdscale command in the MATLAB Statistics and Machine Learning Toolbox. The MDS algorithm served to reduce the dimensionality of the space representing each stimulus shape from potentially up to *n* dimensions, where *n* is the neural population size, to a smaller number of dimensions that capture most of the variance in the data. Such a low-dimensional representation allows easier visualization of patterns within the data. In presenting the MDS results we plot two dimensions. The two dimensions capture 70–80% of the data variance for shape and over 85% of the variance for location. There are eight points plotted in the low-dimensional space resulting from the MDS procedure because there are eight stimuli (either shapes or locations).

##### Stress

We used an error measure called stress to quantitatively compare MDS results from one condition with some other condition (either another MDS result or some aspect of the physical world). The configuration of points in low-dimensional space that results from MDS will have particular positions with respect to each other, but the configuration will be linearly transformed in an indeterminate way, meaning that the configuration as a whole can be linearly scaled, translated, rotated, or reflected. Because we wanted to compare the configuration of points produced by MDS with a set of corresponding points from some other condition, we removed this linear indeterminacy by applying the Procrustes transform (using the procrustes command in the MATLAB Statistics and Machine Learning Toolbox). The Procrustes transform takes two configurations of points and applies a linear transform that minimizes the difference between them, as indicated by the stress error measure:

(1) $$Stress=\sqrt{\frac{\sum_{i}\sum_{j}{\left(d_{ij}^{1}-d_{ij}^{2}\right)}^{2}}{\sum_{i}\sum_{i}{\left(d_{ij}^{1}-〈d_{ij}^{1}〉\right)}^{2}}}$$

In the equation, $d_{ij}^{1}$ is Euclidean distance between points *i* and *j* in the first configuration and $d_{ij}^{2}$ is the Euclidean distance between the corresponding points in the second configuration. The residual stress that remains after the best-fit linear transform is a global measure of how closely the positions of two sets of points match each other.

Stress is therefore a global measure of the difference between two neural response spaces. To measure the statistical significance of that difference, we used bootstrap resampling of the data. We started with a null hypothesis that there was no statistical difference between the data under two attentional conditions (i.e., data from the two conditions were two samples from an identical distribution). To test that hypothesis, we pooled firing rates from individual trials for the two attentional conditions into a single array, shuffled the array, and then split the shuffled array into two subarrays. We then separately did the MDS analysis on these two subarrays and calculated the stress between the MDS results for the two. The MDS analysis was repeated 10,000 times, so we had 10,000 stress values. The *p* value for the hypothesis that the two attentional conditions were significantly different was given by the fraction of resampled stress values greater than the stress value observed in the original data.

## Results

Behavioral response times and error rates for the different tasks have previously been presented in Sereno and Amador (2006; their Fig. 16).

### Single-cell analyses

To allow direct comparisons of our data to much previous work on the neural basis of attention, we first performed single-cell analyses, separately examining responses of each cell to all stimulus shapes (at the most effective location) and to all stimulus locations (using the most effective shape).

#### Attentional gain factors for stimulus shape

**Figure 2.**
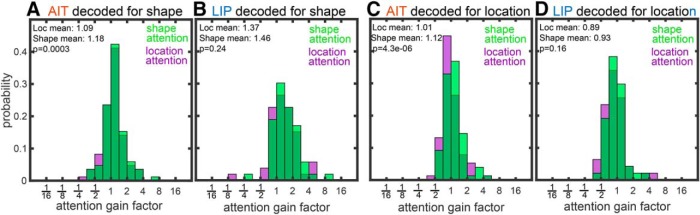
Attentional gain factors associated with the two attention tasks (shape attention task and location attention task). ***A***, ***B***, Attentional gain factors of AIT and LIP neurons when neural responses were decoded for stimulus shape. Gains for the two attention tasks (attention to shape in green and location in purple) were measured relative to neural activities during the passive shape task. ***C***, ***D***, Attentional gain factors of AIT and LIP neurons when neural responses were decoded for stimulus location. Gains for the two attention tasks were again measured relative to neural activities during the passive location task. Significance of the difference between attention tasks was calculated using the Wilcoxon sign rank test.

For each cell we calculated an attentional gain factor for the two attentional tasks relative to the passive shape task. In AIT, there were 85 cells held long enough to obtain sufficient data for all three tasks, while in LIP there were 53 cells. Histograms of these gain factors for AIT are shown in Figure 2*A*, and for LIP in Figure 2*B*. On average, at the single-cell level, attentional demands (both attention to shape or attention to location) increased responsiveness to the different shape stimuli in both AIT and LIP compared to the passive shape task. At the single-cell level, LIP showed greater attentional modulations than AIT (difference between LIP and AIT significant at *p* = 0.010 for the shape attention task and *p* = 0.0047 for the location attention task under the Wilcoxon rank sum test). Noteworthy is that in both brain areas there was a broad range of attentional gain factors for stimulus shape across the population.

#### Attentional gain factors for stimulus location

Analogous to shape, for each cell we calculated an attentional gain factor for the two attentional tasks relative to the passive location task. In AIT, there were 76 cells held long enough to complete the two attention tasks and passive location task, while in LIP, there were 47 cells. Histograms of these gain factors for AIT are shown in Figure 2*C*, and for LIP in Figure 2*D*. In LIP, both attention to shape and to location actually decreased mean responsiveness to different locations compared to the passive task (mean gain factor < 1). In AIT, there was little change in mean responses during attention to location and a slight enhancement for attention to shape compared to the passive task (mean gain > 1). The *p* values for the difference between AIT and LIP was *p* = 0.017 for the shape attention task and *p* = 0.21 for the location attention task under the Wilcoxon rank sum test. In both brain areas, stimulus location shows a broad range of attentional gain factors across the population, as was the case for stimulus shape.

### Population analyses

We refer to neural population representations for a set of stimulus shapes as collectively forming a multidimensional “shape space.” Each shape will have a position in the shape space defined by the population response to that shape. The representational distance from other shapes is defined by the difference in population responses as specified by some metric (in our case, the correlation distance, 1–*r*). Analogously, population representations for a set of stimulus locations collectively form a “location space.” Below we examine attentional effects on the shape space and location space.

#### Response distances of shapes and locations

**Figure 3.**
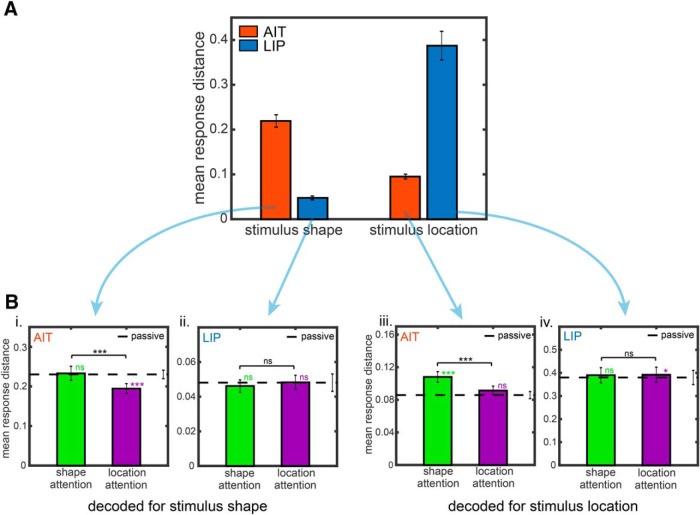
Mean distance between population response vectors. ***A***, Mean distances between population responses pooled over all stimulus shapes or all stimulus locations in areas AIT (red bars) and LIP (blue bars), and also pooled across all attentional conditions. This plots the “3 task average” distances in Table 1, bottom row. ***Bi***, ***Bii***, Attentional effects on mean response distance pooled over all stimulus shapes. The dashed lines indicate the response distances under the passive shape task. ***Biii***, ***Biv***, Attentional effects on mean response distance pooled over all stimulus locations. The dashed lines indicate the response distances under the passive location task. Asterisks indicate statistically significant differences under the Wilcoxon rank sum test. Error bars indicate SE.

For each cortical area, Table 1, columns AIT and LIP under shape space, shows the mean response distances between different stimulus shapes for each of the three tasks (shape attention, location attention, and passive), as well as the average distance collapsed across the three tasks. For each cortical area, Table 1, columns AIT and LIP under location space, shows the mean response distances between different stimulus locations for each of the three tasks, as well as the average distance collapsed across the three tasks. Figure 3*A* (plotting the bottom row of Table 1) illustrates the average distance of the three tasks in area AIT and LIP for different shapes and different locations. These data demonstrate that response distances between shapes were greater (greater representational discriminability between shapes) in AIT than LIP, and furthermore, distances between locations were greater (greater representational discriminability between locations) in LIP than AIT.

**Table 1.**
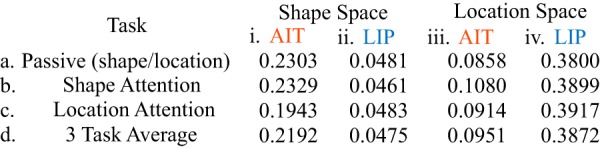
Population response vector distances

i, ii, Mean distances between population response vectors for different stimulus shapes under three tasks, the shape attention task, the location attention task, and the passive shape task. Results for AIT are based on the distance matrices underlying the MDS results in Figure 4. Results for LIP are based on the distance matrices underlying the MDS results in Figure 5. iii, iv, Mean distances between population response vectors for different stimulus locations under three tasks, the shape attention task, the location attention task, and the passive location task. Results for AIT are based on the distance matrices underlying the MDS results in Figure 6. Results for LIP are based on the distance matrices underlying the MDS results in Figure 7.

Figure 3*B* (based on Table 2) breaks down the averages shown in Figure 3*A* according to task, plotting the effect of attention to shape versus attention to location on mean distances for each cortical area. These attentional effects are plotted separately for shape space (Fig. 3*Bi*,*ii*) and location space (Fig. 3*Biii*,*iv*). Interestingly, the attentional condition (attention to shape vs location) did not significantly alter mean distances in LIP in either shape space (Fig. 3*Bii*; Table 2; -4.5%, *p* = 0.08) or location space (Fig. 3*Biv*; Table 2; -0.47%, *p* = 0.31). These data indicate that at the population level LIP responses were relatively constant under changing voluntary attentional demands. In contrast, attentional condition significantly affected mean distances in AIT for both shape space (Fig. 3*Bi*) and location space (Fig. 3*Biii*).

**Table 2.**
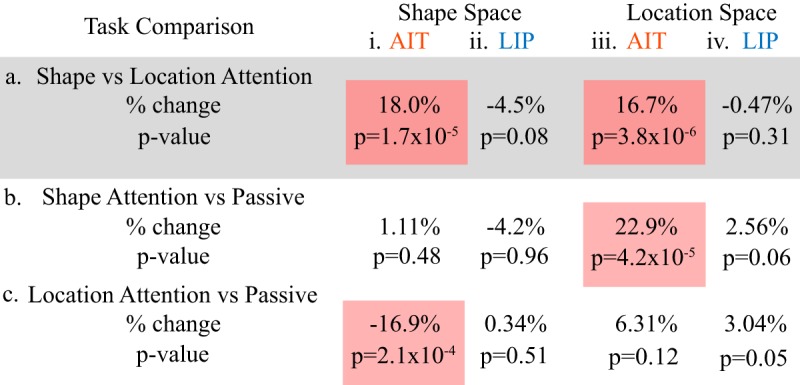
Comparison of population response vector distances under different attentional conditions

a, Shape attention versus location attention. Positive % change values indicate shape attention larger. b, Shape attention versus passive. Positive % change values indicate shape attention larger. c, Location attention versus passive. Positive % change values indicate location attention larger. For each comparison, i, ii shows population response distances between different shapes, and iii, iv shows population response distances between different locations. Red shading shows statistically significant attention effects at the *p* = 0.05 level. In AIT (but not LIP), attention to shape significantly increased response distances to both shape and location, making the shapes and the locations of the shapes more discriminable or distinctive in their neural representations. The comparison between the two attention tasks is highlighted in gray as being of primary interest here.

We next compared the mean response distance for each attentional task (shape or location) with the response distance for its corresponding passive condition (dashed horizontal line in each plot in Fig. 3*B*, with significance indicated immediately above the histogram bar; see also Table 2).

For shape space, when comparing the attentional tasks with the passive tasks, LIP showed little change in mean response distances between different stimulus shapes (no differences; Fig. 3*Bii*; Table 2; -4.2%, *p* = 0.96 for shape attention, and +0.34%, *p* = 0.51 for location attention). Similarly, for location space, LIP showed little change in mean response distances between different stimulus locations (marginal or small differences; Fig. 3*Biv*; Table 2; +2.56%, *p* = 0.06 for shape attention, and +3.04%, *p* = 0.05 for location attention).

AIT showed larger modulations than LIP in mean response distances between the attentional and passive tasks in both shape and location space. Specifically, for shape space (Fig. 3*Bi*; Table 2), attention to location reduced shape response distance or shape discriminability (-16.9%, *p* = 0.0002) but with little change for attention to shape (+1.11%, *p* = 0.48). For location space (Fig. 3*Biii*; Table 2), attention to shape increased location response distance or spatial discriminability (+22.9%, *p* = 0.00004) with little change for attention to space (+6.31%, *p* = 0.12).

#### MDS plots and changes in the organization of shape and location space

In addition to quantifying attentional effects on mean response distances pooled over all stimuli, as in Figure 3, we examined how attention affects relative responses of individual stimuli by plotting results from MDS. Multidimensional representation spaces for stimulus shape are shown for AIT (Fig. 4) and LIP (Fig. 5). These show response distances between individual shapes and their relative positions in shape space. The multidimensional representation spaces for location are shown for AIT (Fig. 6) and LIP (Fig. 7), showing response distances between individual stimulus locations and their relative positions in location space.

**Figure 4.**
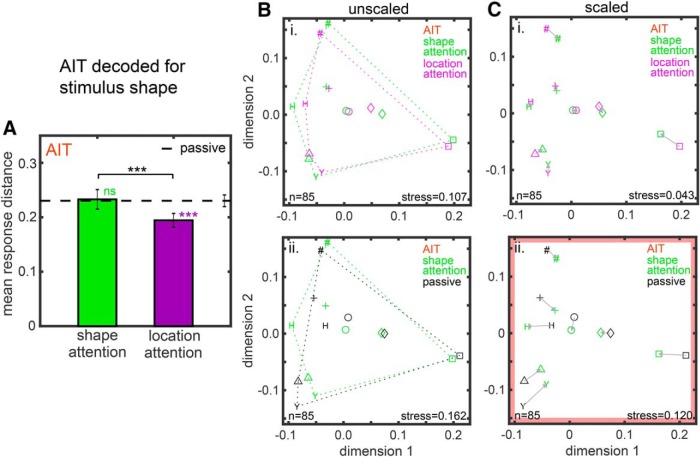
Attentional effects on shape space in AIT based on MDS analysis of neural population responses. ***A***, Mean response distance pooled over all stimulus shapes under the shape attention and location attention tasks (from Fig. 3*Bi*). ***B***, MDS shape space compared under two attentional conditions. ***Bi***, Shape attention task versus location attention task. ***Bii***, Shape attention task versus passive shape task. In both cases, the Procrustes transform was applied to align results from the two attentional conditions. Here, the scaling aspect of the Procrustes transform was turned off to retain differences in mean distance between attentional conditions as seen in ***A***. Dotted lines indicate the convex hull around the eight points in the shape space for each attentional condition. The areas enclosed by the convex hulls gives a rough measure of the relative mean response distance for the two attentional conditions, in accord with ***A***. ***Ci***, ***Cii***, Same as ***B***, except that the full Procrustes transform was performed here, including scaling, to minimize stress between shape spaces under the two attentional conditions. Arrows link corresponding points in the two shape spaces. Significances of attentional differences between scaled shape spaces were examined by bootstrap resampling, and those significant at *p* = 0.05 level have the panel outlined in red.

**Figure 5.**
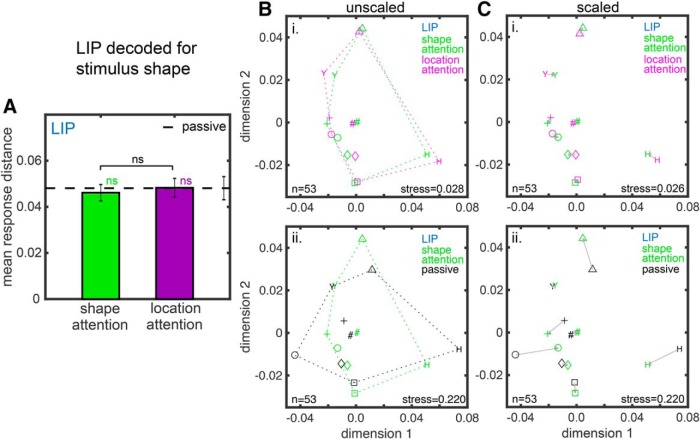
Attentional effects on shape space in LIP based on MDS analysis of neural population responses. Panels are analogous to Figure 4.

**Figure 6.**
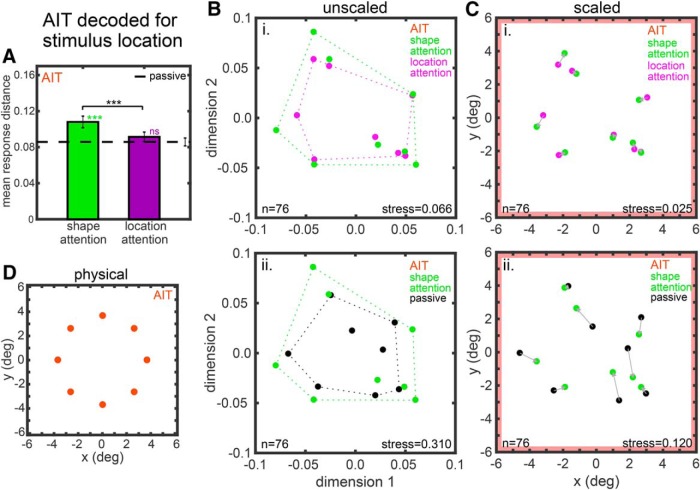
Attentional effects on location space in AIT based on MDS analysis of neural population responses. ***A–C***, Analogous to Figure 4. ***D***, Physical locations of stimuli.

**Figure 7.**
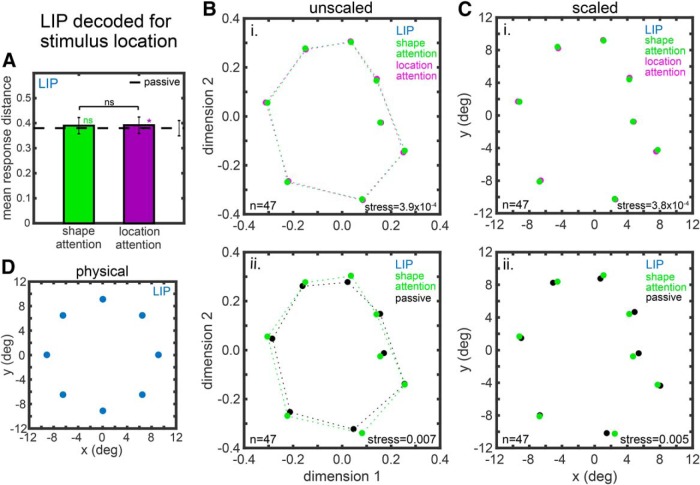
Attentional effects on location space in LIP based on MDS analysis of neural population responses. ***A–C***, Analogous to Figure 4. ***D***, Physical locations of stimuli.

Examining results for shape in AIT further (Fig. 4), Figure 4*A* shows the respective mean response distances, replotted from Figure 3. Figure 4*B* shows attentional effects on MDS shape spaces, comparing the shape attention task versus location attention task (Fig. 4*Bi*) and the shape attention task versus passive task (Fig. 4*Bii*). For each of the two comparisons, the shape spaces have been aligned using the Procrustes transform, but with the scaling aspect of the Procrustes transform turned off. (The Procrustes transform normally aligns two MDS maps using linear scaling, translation, rotation, and reflection to minimize stress between them and thus facilitate comparison of the configuration of points in the two maps.) Because scaling has been turned off, the MDS plots in Figure 4*B* retain information about differences in mean response distance as shown in Figure 4*A*. In each MDS plot in Figure 4*B* the convex hull (enclosing perimeter) around all eight points in the shape space has been drawn (dotted lines). Comparing the sizes (radii) of the convex hulls under two attentional conditions gives a rough indication of the change in mean response distance between the two conditions. In Figure 4*Bi*, the convex hull for the shape attention task is larger than that for the location attention task, consistent with the mean distance histogram in Figure 4*A*. In Figure 4*Bii*, the convex hull for the shape attention task is about the same as the convex hull for the passive task, again consistent with Figure 4*A*. Thus, we see consistency between the mean distance analysis in Figure 3 and the MDS analysis in Figure 4.

Figure 4*C* shows attentional effects on MDS shape representation maps in AIT, as in Figure 4*B*, but this time with the maps for each pair of attentional conditions aligned using the full Procrustes transform, including scaling. Using the full Procrustes transform minimizes the stress between maps. By using the Procrustes transform, we have removed linear effects of attention on the spatial maps, leaving nonlinear effects. By nonlinear attentional effects we mean that attention causes some shape response distances to increase and other shape response distances to decrease or causes response distances to increase or decrease non-uniformly for different shapes. In other words, in addition to linear scaling effects, attention has the possibility of nonlinearly distorting or warping the multidimensional shape representation space. To determine whether the attentional warping of the shape spaces was significant, we performed bootstrap resampling of the data as described in the Methods section. We found the shape spaces for the shape attention task versus location attention task were not significantly different at the *p* = 0.05 level (Fig. 4*Ci*). However, there was a significant difference in the shape attention task versus passive comparison, indicated by a red border around Figure 4*Cii*.

How attention affects the configuration (relative positions) of responses for individual stimuli within the shape space for LIP is shown in Figure 5, with all the conventions as in Figure 4. For LIP there were no significant attentional effects in the MDS maps for either the shape attention task versus location attention task (Fig. 5*Ci*) or the shape attention versus passive task (Fig. 5*Cii*) comparisons (hence no red borders in the panels).

The representation space for stimulus location for AIT is shown in Figure 6 and for LIP in Figure 7. Figures 6*A–C*, 7*A–C* are again analogous to Figure 4. Panel d shows the physical locations of stimuli (this panel has no analog for shape in Figures 4 or 5, because there is no canonical or universally accepted physical shape space). The attentional effects on AIT location space are significant at the *p* = 0.05 level for both the shape attention versus location attention comparison (Fig. 6*Ci*) and the shape attention versus passive comparison (Fig. 6*Cii*), as indicated by the red borders of those panels. In contrast, attentional effects on LIP location space were not significant for either attentional comparison (Fig. 7*Ci*,*ii*).

Table 3 summarizes how attention distorts MDS shape representation spaces (Figs. 4, 5) and location representation spaces (Figs. 6, 7) as measured by stress (Eq. 1). It shows stress values for different attentional task comparisons, as were presented in Figures 4–7*C*, plus the *p* values for the comparisons. The table also includes results for the location attention versus passive comparisons, whose MDS plots were not shown in Figures 4–7.

**Table 3.**
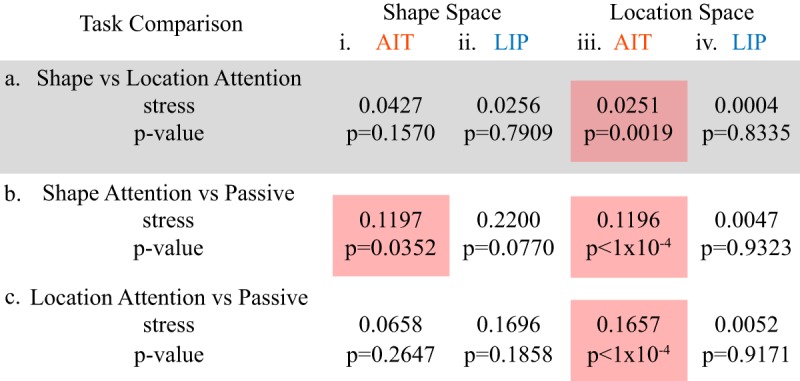
Stress **between pairs of MDS stimulus representation spaces corresponding to different attentional tasks**

Stress (Eq. 1) quantifies global differences between the shape spaces or location spaces under the different attentional conditions. Smaller stress indicates that representation spaces for two tasks are more similar; *p* values of the task differences are based on bootstrap resampling. Red shading shows statistically significant differences at the *p* = 0.05 level. For shape space, results for AIT correspond to MDS plots in Figure 4 and results for LIP correspond to MDS plots in Figure 5. For location space, results for AIT correspond to MDS plots in Figure 6 and results for LIP correspond to MDS plots in Figure 7. The comparison between the two attention tasks is highlighted in gray as being of primary interest here.

Significant attentional task effects at the *p* < 0.05 level are indicated by red shading in Table 3. We see significant tasks effects in both the AIT shape space and AIT location space. None of the attentional task comparisons were significant for either LIP shape space or, interestingly, LIP location space. These findings show that attentional effects can produce changes in the population encoding of individual stimuli such as to shift the relative positions of those stimuli non-uniformly within a multidimensional representation space. In other words, these data show attentional warping of representation spaces. Such warping was substantially larger in AIT than in LIP.

Finally, in Table 4, we see stress values when comparing stimulus locations recovered by MDS with physical stimulus locations. As we have reported previously (Sereno and Lehky, 2011b), stress values were much smaller in LIP than AIT, indicating that stimulus location was represented more accurately by neural population representations in LIP. Further, attention directed to shape or location did not have a major effect on the accuracy with which LIP represented physical locations.

**Table 4.**
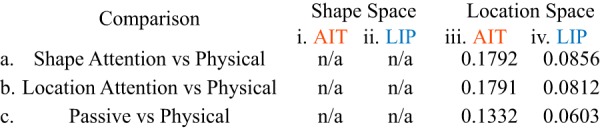
Stress between MDS representational spaces and physical stimulus spaces

Since a canonical physical shape space does not exist, no values are given for shape space. The values for location space indicate the stress between physical locations of stimuli (Figs. 6D, 7D) and MDS results under different attentional conditions.

## Discussion

Studies of attention generally focus on its ability to increase the salience of a target through a variety of means, including competitive effects as well as changes in gain, noise, correlation, and synchrony. All these mechanisms essentially increase the signal-to-noise ratio of the stimulus without indicating whether attention affects the representation or encoding of stimulus shape or location itself. While enhancing salience is certainly an important mechanism for attention, here we demonstrate how attention to stimulus shape or location differentially affects the organization of neural population representation spaces for shape and location in ventral and dorsal cortical brain regions. Such findings indicate that attention may alter population representations of stimuli, and not just enhance a fixed, pre-existing stimulus representation. Most importantly, despite similar attentional gain effects at the single-cell level across the two visual streams, we demonstrate striking differences in attentional effects at the level of population representation within different cortical areas.

Specifically, we report two changes in high-dimensional neural representation spaces due to attention. The first was a general magnification of the representation space, leading to increased discriminability between stimuli because their representations were further apart in the representation space. This increase in discriminability was measured as an increase in distances between population response vectors for different stimuli (Fig. 3). The second was a global, nonlinear warping of the representation space (Fig. 4*C*). Both these effects were much larger in AIT than in LIP.

Previous investigations using fMRI (Jiang et al., 2013; Jackson et al., 2017) or evoked potentials (Rotermund et al., 2009) have suggested that attention can change the representation of stimuli, as shown by increased discriminability between pairs of stimuli or pairs of stimulus categories. However, none of these studies went beyond pairwise analyses to perform a full multivariate analysis over a larger set of stimuli to reveal the global organization of perceptual representation spaces. Çukur et al. (2013) have demonstrated warping of semantic representations using multivariate analysis of fMRI data. This study extends those observations by demonstrating the effect in perceptual representations rather than semantic representations, by demonstrating the effect in populations of individual neurons rather than brain imaging data, and by demonstrating differences in these attentional effects in different cortical areas.

**Figure 8.**
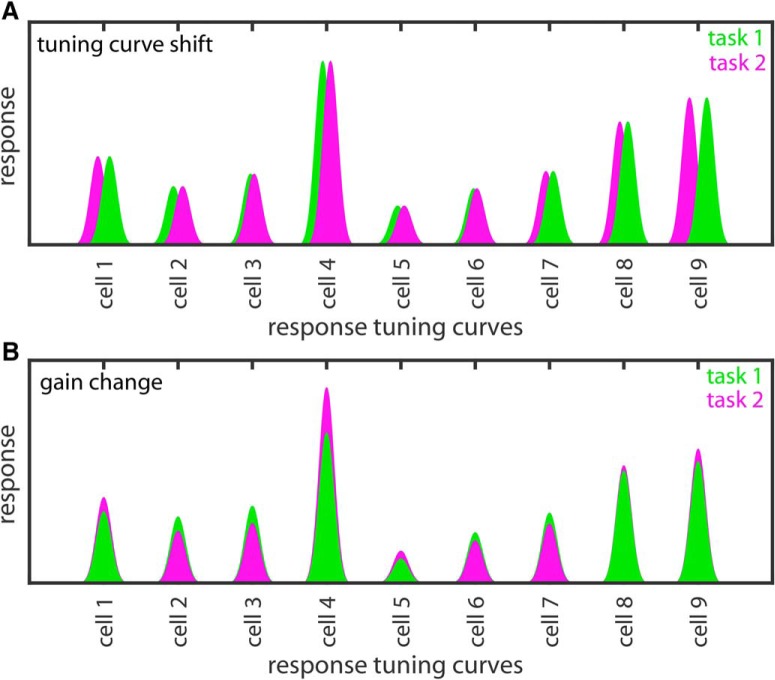
Schematic diagram showing two possible ways how attentional modulations of neural properties across a population could lead to a change in the representation of a stimulus. ***A***, Attention dependent shifts in neural tuning curves. ***B***, Attention dependent changes in neural gain.

Two possible ways that attention could change the population representation of a stimulus are: (1) shifting tuning curves or (2) changing gains (Fig. 8). As reviewed in the introduction, there is evidence for both attentional shifts in tuning curves and gain changes at the single-cell level. Note that either non-uniformity in attentional gain across a population or non-uniformity in tuning curve shifts can cause warping in a representation space. The relative extent to which these two mechanisms actually make significant contributions to changes in representation caused by attention remains to be investigated.

At the single-cell level, attentional effects were apparent in both AIT and LIP and comparable in the two brain areas, as seen in the attentional gain factors in Figure 2. Observation of such single-cell attentional effects in both visual streams is consistent with previous reports, including those in AIT (REFS here) and those in LIP (REFS here), among many others. (Desimone, 1998; McAdams and Maunsell, 1999; Reynolds et al., 2000; Williford and Maunsell, 2006) in AIT and (Treue and Maunsell, 1996; Ben Hamed et al., 2002; Martínez-Trujillo and Treue, 2002; Womelsdorf et al., 2008; Ibos and Freedman, 2014, 2016) in LIP, among many others. None of these studies examined attentional effects at the population level, as we have done here. Why attentional modulations at the single-cell level do not necessarily translate into strong attentional modulations at the population level may be an interesting topic for future modeling.

We found that attentional effects at the population level were substantially weaker in LIP than AIT. That was true for both population vector distance (Table 2) and the MDS analysis (Table 3). Although at the population level we found that all the significant attentional task comparisons (shape attention vs location attention or attention vs passive) were in AIT and none in LIP at the *p* = 0.05 level, some LIP comparisons were borderline for significance (in the range *p* = 0.05–0.07). Given that some population attentional effects in LIP were marginal, we do not feel our findings justify stating that population attentional effects occurred only in AIT but not LIP. It is possible that under different conditions, perhaps boosting signal or reducing noise, one would see small but significant attentional effects in LIP as well. Nevertheless, our findings do indicate that under identical task conditions there were stronger attentional effects at the population level in AIT than in LIP.

As might be expected, in AIT attention to shape versus attention to location increased response distances between shapes, making them more discriminable or distinctive (Fig. 3*Bi*). Counterintuitively, however, in AIT attention to shape versus location also increased response distances between locations, indicating different spatial locations were more discriminable as well (Fig. 3*Biii*). In contrast, attention to shape versus location did not increase response distances (discriminability) between locations in LIP, making LIP population changes an unlikely source for the effects in AIT. Instead, these findings raise the possibility that the changes in AIT were specific to AIT and dependent on the object properties themselves. Such an interpretation suggests that both spatial and shape representations as well as attentional effects in one cortical stream are independent of those in the other cortical stream, and are developed, organized, and independently constrained by different goals, attentional demands, or functions. These findings suggest that attention effects might better be conceptualized as more local, non-homogenous systems rather than a centralized system (cf. Patel et al., 2015).

The spatial relationships between objects are important for defining scenes, and that information is present in AIT (Hayworth et al., 2011; Kim and Biederman, 2011). Small spatial translations in objects or scenes are often important and meaningful, as is the spatial arrangement of parts within objects (e.g., open vs closed lock or window). Our findings suggest that attending to shape in AIT, in addition to enhancing shape representations, may directly enhance the representational discriminability or distinctiveness of different spatial arrangements of stimuli, such as objects within scenes or parts within an object.

Our data had a number of limitations. First, population data were recorded serially, one cell at a time, using single electrodes, rather than in parallel with multielectrodes. Thus, cells recorded at different times were likely to be modulated by randomly varying states of general arousal in the monkey. The effect of constructing populations from serial recordings can be considered equivalent to adding uncorrelated noise to our data across different attentional conditions. Second, we had to estimate some missing values to make the data mathematically suitable for population analyses. The existence of estimation errors was again equivalent to adding uncorrelated noise to our data. We believe the net effect of both these limitations was to make our data noisier, thereby indiscriminately reducing statistical *p* values under all conditions and making it less likely to observe significant effects rather than make it more likely. Nevertheless, we did observe highly significant *p* values under some conditions, and just barely missed significance in a few other conditions.

Task difficulty is a factor that can affect neural responses (Spitzer et al., 1988). Previous analysis of behavioral data (Sereno and Amador, 2006) shows significant increases in response time in the shape attention task compared to the location attention task under identical stimulus conditions. Hence, it is possible that there are differences in task difficulty across the two attention tasks (e.g., shape attention task more difficult than location attention task). However, in these data it is unlikely that differences in task difficulty are driving observed changes in stimulus encoding at the population level. First, task difficulty differences were identical regardless where we were recording from and cannot in themselves account for cortical area differences. Second, the passive tasks are much simpler tasks than either attention task. Despite that, in AIT results from the passive task either aligned with the “more difficult” shape task for shape space (Fig. 3*Bi*) or with the “less difficult” location task for location space (Fig. 3*Biii*). These findings are difficult to explain by task difficulty. Finally, in LIP, there is not much difference among any of the tasks regardless of task difficulty.

We measured attentional effects during the task sample period when there was only a solitary stimulus present on an otherwise blank screen (aside from the fixation spot). Possibly such a singleton stimulus may have resulted in a ceiling effect for attentional modulations in LIP, contributing to weak attentional modulations under different attentional conditions in the population analyses of LIP data. However, looking at the single-cell data, there was nothing in the gain factor plots (Fig. 2) to suggest that the singleton stimulus resulted in a ceiling effect for attention in LIP but not AIT. In both areas there was a wide range of gain factor values. Also, in both animals, there were significant behavioral differences under different attention tasks (Sereno and Amador, 2006). Nevertheless, further work is needed to examine the effects of distractors on population coding and cortical area differences during attentional modulations.

Our analyses have focused solely on attentional effects on mean firing rates. Attention also has effects on other neural firing statistics. As was mentioned in the introduction, those effects include a decrease in the Fano factor, a decrease in noise correlations between neurons, and an increase in correlated oscillations across a population. We can qualitatively estimate the effects those other statistical modulations had on our results.

The effect of Fano factors was already implicitly included in our MDS analysis, which involves trial-by-trial bootstrap resampling of the data to calculate *p* values. Lower Fano factors, as occurs during attention, would lead to more precise estimates of mean firing, and in turn larger *p* values for differences between attentional conditions in the MDS analysis.

Noise correlation between neurons decreases during attention. Looking at neural responses under two attentional conditions meant looking at neural responses during two different trials. Anything that reduced noise correlation between trials would increase the difference between population activities during the two trials for the two attentional conditions. Therefore, *p* values for differences between attentional conditions in the MDS analysis would again have increased.

Attention-related correlated oscillations within the cortical inputs to AIT or inputs to LIP would be expected to increase firing rates in AIT or LIP due to increased effectiveness of synaptic transmission from those input areas (Salinas and Sejnowski, 2001; Azouz and Gray, 2003). Correlated oscillations within AIT or LIP themselves would further increase the effectiveness of input synaptic transmission (Fries, 2015; Ni et al., 2016). Any increased firing rates in AIT and LIP caused by attentionally induced correlated oscillations would have already been included in our analyses, leading to increased *p* values when comparing attentional and passive conditions.

Warping of a representation space is the expected consequence whenever attentional modulations are non-uniform across a population. While we have qualitatively estimated the effect of various firing rate statistics on the mean size of attentional modulations, it is more difficult to predict their effects on the variance of attentional modulations across a population, which is the relevant parameter for determining warping.

Weaker attentional modulations in LIP at the population level might be understood functionally in the context of the distinction made by Goodale and Milner (1992) between vision-for-perception in AIT and vision-for-action in LIP. Perhaps dorsal representations of shape and location are more resistant to cognitive modulations, such as attention, to the extent that these representations are engaged in visuomotor control and therefore need to accurately guide interactions of the body with the physical world. Reports that visuomotor control of grasping is resistant to visual illusions (Haffenden et al., 2001; Aglioti et al., 1995), although controversial (Carey, 2001; Franz, 2001), are consistent with greater cognitive impenetrability in LIP visual processing. Our findings of reduced attentional modulations of population representations in LIP compared to AIT, including alterations of either the representational discriminability of different stimuli or the warping of representation spaces, are consistent with the idea of relative cognitive impenetrability in LIP.

In conclusion, we demonstrate that attention is capable of producing changes in stimulus representations at the population level, going beyond well-studied effects of attention in increasing the salience (or signal-to-noise ratio) of stimuli. We found the strongest population effects of attention in altering representations to be in AIT, where we found that attention can alter both shape and spatial representations, enhancing stimulus discriminability or distinctiveness and also warping representational geometry. In contrast, in LIP, we found relative stability of both shape and location representations under varying attentional conditions. We suggest that reorganizations in the geometry of multidimensional representational spaces in particular brain areas may be an important effect of attention. Our findings suggest that attention will modulate the multidimensional representational spaces in AIT in complex ways that may expand or shrink certain regions of object space or location space to enhance performance of the task at hand (for example grouping stimuli in different ways during a particular categorization task). Together these findings demonstrate that single-cell findings do not necessarily relate to what is happening at a population level (as both areas show comparable single-cell modulations whereas one area shows substantial and stronger changes in the representations of shape and space compared to the other). Our findings also demonstrate that attention can have independent and locally-specific effects on different cortical areas. Importantly, we demonstrate distinctive and counterintuitive attentional effects in cortical representations, with attention to shape improving the spatial representation in AIT. Finally, these findings reveal plasticity in both population representations of shape and location in AIT due to cognitive factors, findings that help link cellular attentional modulations more tightly with behavior and which are certain to stimulate further investigations.

## Synthesis

Reviewing Editor: Valina Dawson, Johns Hopkins School of Medicine Decisions are customarily a result of the Reviewing Editor and the peer reviewers coming together and discussing their recommendations until a consensus is reached. When revisions are invited, a fact-based synthesis statement explaining their decision and outlining what is needed to prepare a revision will be listed below. The following reviewer(s) agreed to reveal their identity: Peter Janssen

Both reviewers have identified interesting aspects of the current manuscript submission. They note that this is a new analysis of existing data recorded in the macaque inferotemporal cortex and in area LIP. The authors apply a multidimensional scaling (MDS) analysis on the responses of the neurons in these two areas while the monkey is either attending to shape or to location, or during passive fixation. The results demonstrate that the neural representation of shape and location changes in IT cortex, but not in LIP. MDS is an interesting way to look at neural representations at the population level. MDS has been used before to visualize shape representations in neural space, both in the dorsal and in the ventral stream, (several studies that deserve to be cited: Murata, Romero, Vogels), but not so much on data acquired during attentional tasks. The overarching posed by your work is of interest to the field of visual integrative neurosciences, namely, moving from the single cell responses to shape or location, as classical done, but rather from the perspective of the multidimensional population representation. The statistical and mathematical methods used are state of the art methods.

While the reviewers find your question interesting they raised several concerns that need to be addressed.

Major Points:

1. Interpolation of shape attention modulation: For each cell, only a subsample of objects is presented to the monkey in the active task. The attention modulation of the unpresented objects is approximated by a multiplicative gain factors extracted from the active task (page 14, line 279).

2.Interpolation of spatial attentional modulation: Again, for each cell, only a subsample of location is presented to the monkey in the active task, not necessarily matching the locations probed during the passive task (so as to match the active task with the visual RF position). A spline interpolation between the eight locations in the passive location task was performed to estimate passive location task responses at the three attention task locations (page 16, line 325).

3. While these interpolation methods can be useful to describe average single neuron responses, using them to address population representation changes is very questionable. Attentional changes in the dimensions identified by the MDS are expected to be driven by both major changes in some neurons and more subtle but systematic changes in other neurons. The authors need more convincing evidence that the conclusions drawn are not due a combination of idiosyncratic biases in the original dataset (e.g. , differential eccentricity or location sampling in the active task, in each area biasing the spline interpolation), and multidimensional properties arising (or not arising) due to the above described approximations of attentional modulation.

4. There are major limitations to the dataset which would have been more convincing had these analyses been performed on actual neuronal responses sampling at the locations of interest and on the entire shape sample. MDS is a method that integrates both mean response information and noise distribution information. The impact of uncorrelated noise information needs to be quantified. And that are two sources of uncorrelated noise: (1) The data has been collected on independent recording sessions. Given the high variability of attention information from trial to trial and session to session, investigating warping in these conditions is a strong approximation to whatever really takes place. (2) Any warping of populational representations is expected to result from shared signal and noise mechanisms at the single trial level. The fact that the data are not simultanesous multielectrode recording is also a limitation in this respect and should be noted.

5. Throughout the manuscript, MDS is associated with information decoding from the neuronal population. This is however an approximation, as no measure of confusion is produced. I would suggest the authors to remove all references to decoding, or perform actual decoding inferences to complement the MDS analysis.

6. The absence of effects in LIP is very intriguing. Could this be due to the fact that the tasks were easy to perform? Actually, no information is provided on the behavioral performance of the monkeys on the different tasks, for each of the recording subsets. An alternative interpretation is that since LIP is involved in bottom-up attention, therefore a single shape appearing on the display (as a sample) already engages attention maximally. This could be the reason why there were no significant effects in LIP. With a more complex task (for example more distractors), it might be possible to measure attention effects in LIP as well.

7. Please add to the discussion the additional effects that (mean response, Fano factor, noise correlations, synchronization etc.) and other changes are expected to have on the population representations of shape and location in addition to warping of average representations.

8. The discussion of the absence of effects in LIP is extremely short (page 30, line 644) and is quite counter intuitive, in view of the dynamic reshaping of visual receptive field shape in the dorsal pathway (e.g., MT, Womelsdorf et al; LIP, Ben Hamed et al.) under diverse influences (and not unlike what has been described in the ventral pathway, eg, in V4, (Tolias et al.), corroborating the evidence that perceived distances change under the influence of attention (see for example, Wardak et al, 2011).

Minor:

Figures:

1. Figure 2: to make this figure easier to interpret, could the authors add task-related icons n the top of each of the histograms, to help the reader visually understand the presented data.

2. Figure 2: the overlay of the green and red colors is not easy to read. These colors should not be used together in the same figure.

3. How to reconcile these observations with the Ibos and Friedman LIP observations in feature selective tasks (2014, 2016).

4. Figure 3: can a 95% confidence interval be represented on these plots?

Text:

Discussion, line 655: Instead, these findings: this is an overstatement.

Line 44 ‘breakthrough’ is a bit exaggerated, since MDS has been used before to look at neural representations.

Line 47-129 The introduction can be shortened perhaps by significantly rewriting or removing the first paragraph

Line 212 Did the authors record mainly in LIPd, in LIPv or in both?

Line 245 The data came from the sample period. This means that there was only one stimulus on the screen, which is important for the interpretation.

Line 380 correlation distance. Were the results the same when MDS was applied on the actual average responses of the neurons?

Line 433-440 Very lengthy, can be dropped I believe.

Line 464: If a finding is not significant it is better and more straightforward to just state that the enhancement was not significant (rather than first saying there was a slight enhancement and a few lines later that this was not significant).

Line 550: ‘nonlinearly distorting or warping ...shape space’ Again this refers to the nonsignificant effect in IT cortex. This should be removed.

Line 565 ‘physical locations of the stimuli’ Please indicate the positions with numbers on all panels.

Line 671-693: Very long section, which is not essential and can therefore can be removed.
